# Appraisal of cowpea (*Vigna unguiculata*) hay as a replacement for noug seed (*Guizotia abissynica*) cake in the ration of Gumuz lambs in Ethiopia

**DOI:** 10.1002/vms3.617

**Published:** 2021-08-26

**Authors:** Esubalew Shitaneh, Bimrew Asmare, Aemiro Kahliew, Habtie Arega, Ayele Abebe

**Affiliations:** ^1^ Pawe Agricultural Research Center Ethiopian Institute of Agricultural Research Pawe P.O. Box 25 Ethiopia; ^2^ Department of Animal Production and Technology Bahir Dar University Bahir Dar P.O. Box 5501 Ethiopia; ^3^ Holetta Agricultural Research Center Ethiopian Institute of Agricultural Research Holetta Ethiopia; ^4^ Debre‐Birhan Agricultural Research Center Amahara Regional Agricultural Research Institute Debre‐Birhan Ethiopia

**Keywords:** body weight, cowpea Sewinet variety hay, Gumuz lamb, hay, noug seed cake

## Abstract

**Background:**

The objective of this study was to evaluate the replacement value of cowpea (*Vigna unguiculata*) hay for noug seed (*Guizotia abissynica*) cake on dry matter (DM) and nutrient intake, nutrient digestibility, body weight change, carcass characteristics and economic feasibility of the feeding regime of Gumuz lambs.

**Methods:**

The study was conducted using 25 yearling intact male lambs with an initial body weight of 18.26 ± 0.63 kg (mean ± SD).The lambs were assigned into five treatments: (T1) 272.3 g noug seed cake (NSC); (T2) 200.73 g NSC + 105.55 g cowpea Sewinet variety hay (CSH); (T3) 134.3 g NSC + 211.86 g CSH; (T4) 66.31 g NSC + 313.79 g CSH and (T5) 417.98 g CSH on DM basis. The experiment was conducted using a randomized complete block design (RCBD), and lambs were blocked based on their initial body weight. The feeding trial was conducted for 90 days followed by 7 days of digestibility trial. Natural pasture hay was treated with molasses solution for improvement of palatability and digestibility and offered for all experimental lambs in ad libitum.

**Results:**

The crude protein (CP) contents of natural pasture hay (NPH), CSH and NSC were 4.99, 18.31 and 36.5%, respectively. The total DM intakes of lambs (*P* < 0.05) increased at higher levels of CSH supplementation. The CP intake decreased with increasing levels of CSH supplementation. Digestibility of DM, organic matter (OM), neutral detergent fiber (NDF) and acid detergent fiber (ADF) was improved by a higher level of CSH supplementation but CP digestibility was not affected by supplementation of CSH. The average daily gain (ADG) showed significant difference (*P* < 0.05) among treatments. Similarly, the feed conversion efficiency (FCE) was significantly different (*P* < 0.05) among treatments; higher FCE was recorded at T1 but lower was at T3 and T4. The hot carcass weight (HCW) showed significant difference (*P* < 0.05) among treatments; higher value was observed in T5 (9.36 kg), but the smallest value was recorded for lambs on the T3 (7.36 kg). The higher dressing percentage (DP) on empty body weight basis was recorded in three treatment (T1 = T4 = T5) groups compared with (T2 > T3) treatments. Significantly higher (*P* < 0.05) rib‐eye area was achieved at a high level of CSH and sole NSC supplemented lambs. The economic feasibility showed that the highest profit was achieved in T5.

**Conclusion:**

From the present study, it was concluded that CSH could be used for supplementation of protein source feed for body weight gain for Gumuz lambs by replacing high‐cost concentrates in a native hay‐based basal diet. As a final point, CSH supplementation was recommended as replacement of NSC at 417.98 g CSH (T5) for better biological performance as well as economic value.

## Abstract 2

The research is based on a result that replaces commercial supplements with a new variety of legume leaf as ruminant feed. The finding is novel to mitigate protein malnutrition in growing animals in the tropics.

## 1 INTRODUCTION

Ethiopia has huge livestock population with diversified functions such as food production, assistance in overall agricultural production and processing at the household level (CSA, 2019). The contribution of livestock in the national economy was estimated at 15%–17% of gross domestic product (GDP) and 35%–49% of the agricultural GDP provided through export commodities like live animals, hides and skins to earn foreign exchanges (Getu, 2015). The country receives annually on average about 105 million US dollars from the export sales of live sheep and mutton (ESPS‐LMM 2011). Though sheep in the country have much relevance, their productivity is below their potential; for instance, the average carcass weight of Ethiopian sheep and goats is 10 kg, and an estimated meat production is 3.5 kg per sheep per year in the population (Hirpa & Abebe, 2008). These values are much lower than those of neighboring countries such as Sudan, Somalia, Djibouti and Kenya, which, respectively, produce 13, 13, 12 and 13 kg/head (Sebsibe, 2008). Inadequate feed supply and its poor quality are the major problems for low productivity of sheep (Deresse et al., 2014).

To tap the potential of sheep, feed resources must be improved in quality (CSA, 2019; Shapiro et al., 2015). Supplementation of sheep with protein sources is one of the strategies that improve the efficiency of utilization of available roughage feed resources (Hailecherkos et al., 2020; Obeidat et al., 2020). Noug seed (*Guizotia abyssinica*) cake is one of the commonly utilized protein sources for supplementation of sheep in Ethiopia. However, this supplement feed is becoming limited under smallholder farmers due to insufficient supply and escalating price, which calls to look for other cheap and accessible alternative protein supplements. Among the options, forage legumes which can be produced at local conditions is one of options to improve the productivity of sheep (Hailecherkos et al., 2020; Makuriaw & Asmare, 2018). In the study area, cowpea hay was indicated as a potential protein source used as supplementary feed for small ruminants (Gebrekidan et al., 2019). Agzaet al. (2012) suggested that cowpea (*Vigna unguiculata*) Sewinet variety is one of the best adaptable, short‐duration, high‐forage biomass and nutrient‐rich forage variety, but their nutrient utilization on performance of different animals is not yet determined. However, the potential of cowpea Sewinet variety hay (CSH) as a feed supplement has not been utilized due to various reasons, such as the lack of awareness on the utilization of fodder crops. Assumption of the study was that CSH is substitute NSC as a protein source for fattening of Gumuz lambs. Therefore, this research was conducted to evaluate the replacement of NSC with CSH on nutrient utilization and the overall performance of Gumuz lambs.

## 2 MATERIALS AND METHODS

### 2.1 Forage establishment and feed preparation

Cowpea (*Vigna unguiculata*) Sewinet variety was sown through rain‐fed on well‐prepared land. The land plowing, sowing, fertilizer application, weeding, harvesting, preservation (haymaking) and storage were applied based on recommended practices for the forage (DIAS, 2011). Cowpea Sewinet variety was harvested for hay at 50% flowering, chopped and stored under shade area to prevent nutrient loss due to solar radiation and rainfall. The NPH was harvested at 50% flowering (McDonald et al., 2010). CSH and NPH were chopped to 3–5 cm by the chopping machine to make ready for consumption of lambs and stored on a well‐ventilated concrete floor shade to avoid spoilage and mold development. NSC was purchased from the local market. Molasses were purchased from the Wonji sugar factory and mixed thoroughly with natural grass hay. The experimental feed was mixed to make ISO‐N and ISO‐caloric basis properly before given to the experimental lambs. A 72.53, 63.47, 54.47, 45.18 and 40.65 g of molasses for T1, T2, T3, T4 and T5, respectively, was diluted in water at a ratio of 1:2, and diluted molasses solution was properly mixed with NPH (Valadares et al., 2002). The molasses solution was to improve the palatability and digestibility of feed.

### 2.2 Experimental animals and their management

Twenty‐five intact yearling male Gumuz lambs with a mean initial body weight of 18.26 ± 0.63 kg (mean ±SD) were purchased from the local market. The age of lambs was determined by dentition and asking for birth information from the owners. The experimental lambs were quarantined for 21 days to observe their health condition. During this time, the animals were drenched with anti‐helminthes (Albendazole 300 mg bolus) and sprayed with Amitraz 12.5% with a dose of 1.6 ml per liter water against internal and external parasites, respectively, and vaccinated against common diseases of the area peste des petits ruminants before the beginning of the actual experiment. After the quarantine, the animals were moved to an experimental pen and fed the experimental ration for 15 days of the adaptation period. Then animals were ear‐tagged for identification purposes and due to the weight difference, the lambs were grouped into five blocks based on empty body weight (EBW) which was determined after two consecutive weighing after overnights fasting. Then lambs were randomly assigned to the treatments within the block and placed in an individual pen of the size 0.85 m width × 1.2 m length furnished with feeding trough and watering buckets. The feeding trial was conducted for 90 days followed by 7 days of digestibility experiment. NPH was weighed and offered ad libitum two times a day as a basal diet at a rate of 20% refusal. The supplement feed was offered separately twice a day at 8:00 AM and 4:00 PM in equal proportions. Water and salt lick were available to lambs at all the time. The refusal was collected and weighed every morning before offering the daily ration. After the growth trial, the digestibility study was conducted for seven consecutive days. In the digestion trial, lambs were allowed to adapted fecal bags for four days and a digestibility study was conducted for seven days following adaptation.

### 2.3 Experimental design and treatments

The experiment was conducted using a randomized complete block design (RCBD) with five blocks and five treatments. The lambs were blocked based on their initial body weight which was determined by weighing lambs after overnight fasting. The ration was formulated with different proportions of CSH and NSC, making to meet the requirement of growing tropical lambs given in Table 1. The proportion of CP obtained from CSH was substitution levels of 0%, 25%, 50%, 75% and 100% of NSC for treatments 1, 2, 3, 4 and 5, respectively.

**TABLE 1:** Experimental feeds used for digestibility and growth trail

Treatments	Natural grass hay treated with molasses	Supplement (g/day) on DM basis		Total supplement (g/day/head)
		CSH	NSC	
T1	ad libitum	0	272.3	272.3
T2	ad libitum	105.55	200.73	306.28
T3	ad libitum	211.86	134.3	346.16
T4	ad libitum	313.79	66.31	380.1
T5	ad libitum	417.98	0	417.98

Abbreviations: CP, crude protein; DM, dry matter; NSC, noug seed cake.

### 2.4 Chemical composition of feed refusals and feces

Samples of CSH, NSC and natural pasture hay (NPH) offered and refused were collected daily and pooled over the experimental period for each feed and treatment. The samples of feed offered and refused were dried in an oven to a constant weight at 65^o^C for 72 hours to determine nutrient content. The partially dried samples of feeds and feces were ground using a laboratory mill to pass through a 1‐mm sieve screen size and taken to animal nutrition laboratory for chemical analysis. The dry matter (DM) content of feeds was determined after the drying of another sub‐sample of feeds at 105^o^C in an oven to constant weight. The samples of feed offered, refused and feces were analyzed for DM, ash and nitrogen (N) according to the procedures of AOAC (1990). Crude protein (CP) was estimated as N ×6.25. Neutral detergent fiber (NDF), acid detergent fiber (ADF) and acid detergent lignin (ADL) were analyzed by the method of Van Soest (1994).

### 2.5 Dry matter and nutrient intakes

The daily amount of DM and nutrients offered and the refusal were weighed for each animal and recorded to determine the amount of DM and nutrients consumed as the difference between the offered and refused. A sample of feed offered was taken from batches of feeds and refusals have been collected from each animal across the experimental period and finally pooled for each treatment basis and sub‐sampled for laboratory analysis. Nutrient intake was determined as the difference between the quantity of nutrient offered and refused for each lamb.

### 2.6 Apparent digestibility of DM and nutrients

The apparent digestibility coefficient of DM, OM, CP, NDF and ADF was determined as a proportion of the nutrient intake not recovered in the feces using the following formula:

$$\mathrm{Apparent}\;\mathrm{digestibility}\%\;=\frac{\left(\mathrm{Nutrient}\;\mathrm{intake}\;-\;\mathrm{Nutrient}\;\mathrm{in}\;\mathrm{feces}\right)}{\mathrm{Nutrient}\;\mathrm{intake}}\times 100$$

The digestibility of the supplement feed was determined by difference, which is calculated after knowing the digestibility of the basal diet using the equation of McDonald et al. (2010).

$$\begin{array}{cc} & \mathrm{Digestibility}\;\mathrm{of}\;\mathrm{nutrient}\;\mathrm{in}\;\mathrm{supplement}\;\mathrm{feed}=\mathrm{Nutrient}\;\mathrm{in}\;\mathrm{test}\;\mathrm{feed}\\ & -(\mathrm{Nutrient}\;\mathrm{in}\;\mathrm{feces}\;\mathrm{of}\;\mathrm{mixed}\;\mathrm{diets}-\mathrm{Nutrient}\;\mathrm{in}\;\mathrm{feces}\;\mathrm{of}\;\mathrm{basal}\;\mathrm{feed})\\ & /\mathrm{Nutrient}\;\mathrm{in}\;\mathrm{test}\;\mathrm{feed}. \end{array}$$

Digestible organic matter (OM) contents of treatment feed were estimated by multiplying the OM content by its digestibility coefficient. The metabolizable energy (ME) content of treatment feeds estimated from the digestible OM contents of the feeds using the equation of McDonald et al. (2010) as ME (MJ/kg DM) = 0.0157 DOM, where DOM = g of digestible OM/kg of DM.

### 2.7 Body weight gain and feed conversion efficiency

The initial body weight of each animal was determined by taking the mean of two consecutive weights after overnight fasting. The weight of the animals was measured every 10 days afterward, after overnight fasting. Average daily body weight gain (ADBWG) was calculated as the difference between the final and initial BW divided by the number of feeding days. Feed conversion efficiency (FCE) is the measure of feed utilization how efficient the lambs were converting the feed into meat as indicated in the following formula:

$$\begin{array}{cc} & \mathrm{Bodyweight}\;\mathrm{gain}\;\left(\mathrm{kg}\right)\;=\mathrm{Final}\;\mathrm{body}\;\mathrm{weight}\;\left(\mathrm{kg}\right)\\ & -\mathrm{Initial}\;\mathrm{body}\;\mathrm{weight}\;\left(\mathrm{kg}\right)\mathrm{ADBWG}\;\left(g\right)\;=\mathrm{Body}\;\mathrm{weight}\;\mathrm{gain}\;\left(g\right)\\ & /\mathrm{Number}\;\mathrm{of}\;\mathrm{feeding}\;\mathrm{days}\;\left(90\right)\mathrm{FCE}=\mathrm{ADBWG}\;\left(g\right)\\ & /\mathrm{Daily}\;\mathrm{total}\;\mathrm{DMI}\;\left(g\right) \end{array}$$

### 2.8 Carcass characteristics

The carcass characteristics of experimental lambs were evaluated by slaughtering all lambs in each treatment after overnight fasting at the end of growth and digestibility trials. Slaughter weight (SW) was taken right before slaughter. The lambs were slaughtered by severing the jugular vein and carotid artery with a knife. The blood of each lamb was drained into the bucket and weighed immediately after collection. After the lambs were killed, the skin flayed carefully to avoid the adherence of fat and muscle tissue to the skin, and the skin was also weighed. The entire gastrointestinal tract without the esophagus was removed and divided into two sections as stomach and intestine and weighed with gut fill. During removal of the gastrointestinal tract mesenteric fat was separated carefully. Internal organs, lung, heart, kidney, liver, spleen, pancreas, genital organ, gall bladder and other parts like head, visceral fat and feet have been removed and weighed. The weight of the hot carcass was measured after all the offal's properly removed from the carcass. Based on the feeding habit of the people in the area, edible and non‐edible offal was identified and recorded. The total edible offal components were taken as the sum of blood, heart, head, liver, kidney, tail and visceral fat. The non‐edible offal component was taken as the sum of skin with feet, genital organ, and lung with trachea, spleen, gall bladder and gut fill. The total usable product was taken as the sum of HCW, skin and total edible offal component. To measure the rib‐eye‐area of the carcass loin part was partitioned into fore and hindquarters between the 11th and the last ribs. The cut ribs were chilled for 12 hours in the deep refrigerator and the rib‐eye area in cm^2^ was measured at the 12th and 13th rib sites. The cross‐section of the rib‐eye muscle was traced first on transparency plastic paper after the loin part is cut between the 12th and 13th ribs perpendicular to the backbone. Then the traced transparency paper was positioned on graph paper squares each having an area of 1 mm × 1 mm. The numbers of squares included within the mark were counted for the left and right sides, and the area was computed as the average of the two. Finally, EBW was calculated as the difference between SW and gut content. Dressing percentage (DP) was determined as the proportion of HCW to slaughter body weight (SBW) or EBW.

### 2.9 Statistical analysis

Data on feed intake, digestibility and carcass characteristics were analyzed by analysis of variance (ANOVA) using the GLM procedure of the statistical analysis system (SAS) version 9.3. The difference between treatment means was separated using the least significant difference test.

Thestatisticalmodelwas:Yij=μ+Ti+βj+εij,

where *Yij* is the response variable (an observation in *i* treatment and *j* block); μ is the overall mean; *Ti* is  *i*th treatment (test diets) effects; *βj* is the *j*th block effect (initial body weight effect); and *Ɛij* is  the random error.

## 3 RESULTS

### 3.1 Chemical composition of the experimental feeds and refusals

The chemical composition of experimental feeds and refusals of the experiment is shown in Table 2. The CP content (4.99%) of NPH used in the current study was below the threshold level of (7%) of CP for best microbial activity in the rumen that can support the maintenance requirements of the animals. Hence, the NPH used in this experiment can be characterized as lower quality feed in terms of its CP content. The current NPH refusal had higher NDF, ADF and ADL values than the hay offered. This may be because experimental animals selected more edible portions of the basal diet and left the more fibrous parts (such as stems) of the grass which has higher fiber (NDF, ADF and ADL) fractions. Generally, DM, OM and CP content of all experimental feed offered was greater than feed refusal whereas in T2, T3 and T4 the NDF, ADF and ADL percentage was the reverse. Refusals mainly constituted stem parts of the feed because the stem of the CSH contained higher lignin content. The CP content of CSH offer was higher than refusal, but the NDF, ADF and ADL were contrary to CP content.

**TABLE 2:** Chemical composition of the experimental feed offered and refusals

Feed samples	Chemical composition of feed on DM%						
	DM	OM	Ash	CP	NDF	ADF	ADL
Experimental feed offered							
T1 (100% NSC)	95.89	76.01	23.99	36.5	44.71	28.38	8.36
T2 (75 NSC:25 CSH %)	94.24	81.12	18.88	29	47.26	32.54	7.9
T3 (50 NSC:50 CSH %)	94.58	80.31	19.69	26.19	46.01	31.81	7.28
T4 (25 NSC:75 CSH %)	93.39	83.34	16.66	20.75	47.4	32.71	6.67
T5 (100% CSH)	93.3	80.48	19.52	18.31	43.02	31.57	5.99
MTNPH	90.57	89.61	10.39	4.99	63.44	40.37	5.56
Feed refusals							
T1 MTNPH	90.03	90.30	9.7	3.53	67.88	43.55	6.37
T2 MTNPH	89.77	91.05	8.95	3.98	68.85	44.89	6.71
T3 MTNPH	89.76	90.76	9.24	4.16	70.33	45.78	6.81
T4 MTNPH	89.61	89.41	10.59	4.62	67.91	43.29	6.65
T5 MTNPH	89.87	89.76	10.24	4.6	67.11	44.18	6.67
CSH	93.63	82.57	17.43	14.19	52.32	45.39	8.13

Abbreviations: ADF, acid detergent fiber; ADL, acid detergent lignin; CP, crud protein; CSH, cowpea Sewinet hay; DM, dry matter; MTNPH, molasses treated natural pasture hay; NDF, neutral detergent fiber; OM, organic matter.

### 3.2 Dry matter and nutrient intakes

The DM and nutrient intakes of Gumuz lamb‐fed molasses‐treated NPH and supplemented mixture of CSH and NSC are given in Table 3. Statistically, there was no significant difference on the basal diet DM intake (*P* > 0.05) among treatments. However, numerically higher basal diet DM intake was recorded by T1‐, T2‐ and T5‐supplemented groups compared with T3 and T4 treatments. Apart from T4 and T5 other treatments group were consumed all supplements offered feeds without any refusals. The total DM and OM intakes were significantly different (*P* < 0.05) among the supplemented groups in which the daily total DM intake increased as the level of CSH supplementation increased.

**TABLE 3:** Effect of replacement of NSC with different level of cowpea (Vigina unguiculata) Sewinet variety hay on daily feed intake of Gumuz yearling lambs fed MTNPH as a basal diet

Parameters	Treatments						
	T1	T2	T3	T4	T5	SEM	SL
Feed DMI (g/day)							
NPH DMI	552.73	544.55	517.96	511.53	544.69	43.94	NS
Supplement intake	272.3^e^	306.24^d^	346.04^c^	376.04^b^	400.7^a^	6.48	***
TDMI	825.04^c^	850.79^b^, ^c^	864^b^, ^c^	887.58^[Link]^, ^b^	945.39^a^	43.8	**
TDMI (g/kgW^0.75^)	78.09	81.82	83.98	84.8	87.25	4.86	NS
TDMI (%BW)	3.55	3.74	3.86	3.88	3.94	0.26	NS
Nutrient intake (g/day)							
CPI	126.97^a^	125.01^a^	121.16^[Link]^, ^b^	111.46^b^, ^c^	100.54^c^	8.67	*
OMI	812.6^c^	839.32^b^, ^c^	861.73^b^, ^c^	875.75^[Link]^, ^b^	931.94^a^	43.34	*
NDFI	464.5^b^	481.9^b^	497.2^[Link]^, ^b^	491.1^[Link]^, ^b^	526.5^a^	27.7	*
ADFI	291.3^c^	307^b^, ^c^	315.6^[Link]^, ^b^	313.9^[Link]^, ^b^, ^c^	336.2^a^	17.7	**
ADLI	50.9^b^	54.1^b^	59.7^a^	60.2^a^	64.1^a^	3.4	**
ME(MJ/kg)	5.7^c^	5.96,^b^, ^c^	6.1^b^	6.21^b^	6.68^a^	0.24	**

Abbreviations: ADFI, acid detergent fiber intake; ADLI, acid detergent lignin intake; CPI, crude protein intake; NDFI, neutral detergent fiber intake; NP, natural pasture; NS, non‐significant; NSCI, noug seed cake intake; OMI, organic matter intake; SEM, standard error of mean; SHI, Sewinet hay intake; SL, significance level; TDMI, total dry matter intake.

^a^ Mean values in a row with different superscripts differ significantly.

^b^ Mean values in a row with different superscripts differ significantly.

^c^ Mean values in a row with different superscripts differ significantly.

^d^ Mean values in a row with different superscripts differ significantly.

^e^ Mean values in a row with different superscripts differ significantly.

*** Significant at alpha 0.001.

** Significant at alpha 0.01.

* Significant at alpha 0.05.

The OM, CP, NDF, ADF and ADL intakes of experimental lambs were significantly different (*P* < 0.05) among treatments. The daily CP intake was significantly higher (*P* < 0.05) in T1 and T2 and ranked increasing order in CP intake as T1 = T2 > T3 > T4 > T5. This is associated with the bulky nature of the CSH when it substituted NSC at a higher level (75% and 100%). The estimated ME intake was significantly different (*P* < 0.05) among the treatments. The ME intake ranked the supplemented group as T5 > T4 = T3 = T2 = T1.

### 3.3 Dry matter and nutrient digestibility

The values for DM and nutrient digestibility and digestible nutrient intake of Gumuz lambs fed on basal diet offered molasses‐treated NPH and supplemented with CSH and NSC mixture are presented in Table 4. A significant difference (*P* < 0.05) was observed among treatments in terms of DM, OM and ADF digestibility in the current study. However, there was no significant difference (*P* > 0.05) on CP digestibility as well as digestible CP intake among treatments. The higher DM and OM digestibility observed in sole CSH‐supplemented sheep might be due to a high intake of the supplemented diet, and high concentration of nitrogen in legume forages improves the rate of roughage feed degradation in the rumen. There was no significant difference (*P* > 0.05) on CP digestibility among treatments, but higher apparent digestibility of ADF and NDF was observed in T5.

**TABLE 4:** Effect of replacement of NSC with different level of cowpea (Vigina unguiculata) Sewinet variety hay on DM and nutrient digestibility in Gumuz yearling lambs fed MTNPH as a basal diet

Parameters	Treatments					SEM	SL
	T1	T2	T3	T4	T5		
Digestibility (%)							
DM	57.27^c^	60.49^b^, ^c^	64.34^[Link]^, ^b^	67.46^a^	70.89^a^	4.95	**
OM	57.36^c^	62.18^b^, ^c^	63.86^b^	66.62^[Link]^, ^b^	69.68^a^	3.6	**
CP	58.1	64.64	59.15	60.7	58.06	11.58	NS
NDF	53.65^b^	56.3^b^	59.4^a^	59.5^a^	61.73^a^	1.99	***
ADF	44.9^c^	46.44^b^, ^c^	50.61^b^	50.02^b^, ^c^	57.51^a^	3.83	**
Digestible nutrient intake (g/day)							
DM	370.94^c^	382.1^b^, ^c^	389.75^b^, ^c^	395.75^b^	427.67^a^	14.41	**
OM	359.03^c^	373.01^b^, ^c^	381.23^b^	388.59^b^	417.56^a^	15.29	***
CP	73.7	80.77	71.8	67.5	63.25	15.14	NS
NDF	215.2^c^	224.38^b^, ^c^	235.95^b^	257.26^a^	270.74^a^	10.22	***
ADF	160.26^e^	167.86^d^	173.52^c^	179.02^b^	184.68^a^	3.03	***
ADL	35.5^c^	38.68^b^	39.84^b^	42.76^a^	43.26^a^	1.69	***

Abbreviations: ADF, acid detergent fiber; CP, crude protein; DM, dry matter; NDF, neutral detergent fiber; NS, non‐significant; OM, organic matter; SEM, standard error of mean; SL, significance level.

^a^ Mean values in a row with different superscripts differ significantly.

^b^ Mean values in a row with different superscripts differ significantly.

^c^ Mean values in a row with different superscripts differ significantly.

^d^ Mean values in a row with different superscripts differ significantly.

^e^ Mean values in a row with different superscripts differ significantly.

*** Significant at alpha 0.001.

** Significant at alpha 0.01.

### 3.4 Body weight change, daily gain and FCE

The initial and final body weight, average daily weight gain and FCE of Gumuz lambs fed NPH and supplemented with NSC and CSH and its mixture are presented in Table 5. The current result showed a significant difference (*P* < 0.01) on ADG and FCE among treatment but non‐significant difference (*P* > 0.05) on initial weight and final body weight change among treatments. Higher ADG was recorded in (T1) followed by (T5) in order of T1 > T5 > T2 > T3 = T4. In the present finding, significant difference (*P* < 0.01) was found among treatments in the FCE of Gumuz lambs. Higher FCE was observed in treatment (T1) and lower FCE was observed in treatment (T3 and T4) which might be associated with higher CP intake in T1.

**TABLE 5:** Effect of replacement of NSC with different level of CSH on body weight change, average daily weight gain and FCE of Gumuz yearling lambs fed MTNPH as a basal diet

Parameters	Treatments					SEM	SL
	T1	T2	T3	T4	T5		
IBW (kg)	17.96	18.1	18.23	18.43	18.6	0.63	NS
FBW (kg)	23.52	22.52	22.34	22.68	23.74	1.37	NS
ADG (g/day)	71.32^a^	53.97^b^, ^c^	50.94^c^	51.54^c^	62.68^[Link]^, ^b^	7.98	**
FCE (ADG/DMI)	0.086^a^	0.062^b^	0.058^b^	0.058^b^	0.068^b^	0.01	**

Abbreviations: ADG, average daily gain; FBW, final body weight; FCE, feed conversion efficiency; IBW, initial body weight; NS, non‐significant; ns, non‐significant; SEM, standard error of mean; SL, significance level.

^a^ Mean values in a row with different superscripts differ significantly.

^b^ Mean values in a row with different superscripts differ significantly.

^c^ Mean values in a row with different superscripts differ significantly.

** Significant at alpha = 0.01.

### 3.5 Carcass characteristics

Carcass characteristics were evaluated based on DP on SBW and EBW base, HCW, REA, internal fat deposits, and edible and non‐edible offal.

#### 3.5.1 Slaughter and EBW, HCW and DP

The present values of SBW, EBW, HCW, DP as a proportion on slaughter and EBW basis and rib‐eye muscle area (REA) of the lambs are given in Table 6. Except SBW, the EBW, HCW, DP on slaughter and EBW base REA were significantly different (*P* < 0.05) among treatments. Higher EBW and HCW was recorded in sole Sewinet hay (T5) treatment but the lowest was recorded in T3 (50:50% NSC and CSH), and the other treatment was intermediate between (T5 and T3), but was not statistically significant among each other (T1, T2 and T4). This might be due to higher DM digestibility; this also improved feed intake and the weight gain as well as FCE of lambs. Besides, it is known that leguminous fodder increases protein supply to the host animal by increasing the supply of both degradable and un‐degradable protein and by creating a favorable rumen environment resulting in enhanced fermentation of the basal roughage and thus increased microbial protein synthesis (Osuji et al. 1995). The current finding agrees with the value 17.3 kg EBW and 9.25 kg HCW reported by Gelgelo et al. (2017) for black head Somali sheep supplemented with local brewery by‐product and concentrate mixture. However, lower values reported by Getent et al. (2019), 24.17 kg and 9.72 kg, EBW and HCW for Gumuz lambs supplemented with toasted soybean grain, respectively. The disparity between the current result and previous findings might be associated with the variation on CP digestibility, FCE of lambs and also the breed of sheep used for the experiment. Parameters of DP on EBW base were significantly different (*P* < 0.05) among treatments. A higher value was recorded in treatment (T1 = T4 = T5) groups compared with (T2 > T3) treatments. This trial validated that the sole NSC and higher rate of CSH substitution for NSC positively favored the DP on EBW base of Gumuz lambs due to higher CP content of NSC, better DM digestibility of CSH and better daily weight gain in animals. The present result on the SBW basis was not significantly different (*P* > 0.05) among treatments but higher DP value was observed in T5. The DP on an EBW basis of the current result was significantly different (*P* < 0.05) among treatments. This study achieved a higher DP on SBW and EBW base at a higher level of NSC and SH supplementation.

**TABLE 6:** Effect of replacement of NSC with different level of CSH on carcass characteristics of Gumuz yearling lambs fed MTNPH hay as a basal diet

Parameters	Treatments					SEM	SL
	T1	T2	T3	T4	T5		
SBW (kg)	22.66	22	21.9	22.4	23.46	1.55	NS
EBW (kg)	17.56^[Link]^, ^b^	17.07^[Link]^, ^b^	16.56^b^	17.53^[Link]^, ^b^	17.93^a^	0.97	*
HCW (kg)	8.98^[Link]^, ^b^	8.3^b^, ^c^	7.36^c^	8.7^[Link]^, ^b^	9.36^a^	0.72	*
Dressing percentage on							
SBW base	39.74	37.74	33.92	38.94	40.19	3.37	NS
EBW base	51.12^a^	48.58^[Link]^, ^b^	44.52^b^	49.64^a^	52.3^a^	3.16	*
REA(cm^2^)	7.7	7.32	6.56	6.34	7.6	1.0	NS

Abbreviations: ns, non‐significant; SL, significance level; SEM, standard error of mean.

^a^ Mean values in a row with different superscripts differ significantly.

^b^ Mean values in a row with different superscripts differ significantly.

^c^ Mean values in a row with different superscripts differ significantly.

* Significant at alpha 0.05.

#### 3.5.2 Rib‐eye muscle area

There was no significant difference (*P* > 0.05) on REA among treatments, but higher REA was recorded in sole NSC‐supplemented group and the lowest was recorded in T4 (25:75% NSC and CSH), while similar values were observed in T2, T3 and T5.

#### 3.5.3 Carcass offal

In Ethiopia, religious, traditions, beliefs and cultural differences present a variety of meat consumption behaviors from one locality to the other. Based on above criteria, offal components are referred to as varieties of meat consisting of internal and external organs categorized as edible and non‐edible offal. Heart, liver, empty gut (stomach + small and large intestine), visceral fat, tail, tongue, blood, lung with the trachea, testis and kidneys are considered as edible offal. Although spleen, pancreas, skin, feet, head, genital organ, esophagus, gull and urinary bladder and gut contents are considered as non‐edible offal. In the current study, there was no significant difference (*P* > 0.05) among treatments on edible offal components except tongue. The weight of the tongue was significantly (*P* < 0.05) higher in T5 than in other treatment groups. This might be due to the rumen micro flora boosted for experimental animals fed on sole CSH that improved the development of associated organs. The current finding indicates that there were no significant differences (*P* > 0.05) in total edible offal components among treatments, but the high value was recorded in the high CSH‐supplemented groups (T4 and T5) compared with other treatments.

Non‐edible carcass offal of the experimental lambs are presented in Table 7. A significant (*P* < 0.05) difference was observed among T2, T3 and T4 for spleen and urinary bladder. This finding indicated that the replacement of CSH at 75, 50 and 25 had improved the growth of the abovementioned parameters. However, the present study did not show significant difference (*P* > 0.05) among treatments for gut content, ahead without tongue, skin, feet, genital organ, gall bladder and total non‐edible offal components.

**TABLE 7:** Effect of replacement NSC with CSH on non‐edible carcass offal of Gumuz lambs fed MTNPH as a basal diet

Parameters	Treatments					SEM	SL
	T1	T2	T3	T4	T5		
Head (kg)	1.29	1.38	1.21	1.32	1.23	0.14	NS
Esophagus (g)	37.5	40.5	40.3	46	47.8	7.82	NS
Lung and trachea (g)	317.4	286.6	281.9	324.9	285.3	51.77	NS
Gut contents (kg)	5.1	4.92	5.33	4.86	5.53	0.83	NS
Skin and feet (kg)	2.85	2.81	2.82	2.86	3.1	0.3	NS
Spleen and pancreas (g)	57.7^b^	87^[Link]^, ^b^	72.58^[Link]^, ^b^	93.2^a^	59.76^b^	4.9	*
Genital organ (g)	299.3	286.5	300.5	307.9	318.28	66.3	NS
Urinary and gall bladder (g)	17.2	15.7	27.4	34.5	18.2	14.47	NS
TNEO (kg)	9.97	9.84	10.1	9.86	10.61	1.02	NS

Abbreviations: ns, non‐significant; SL, significance level; SEM, standard error of mean.

^a^ Mean values in a row with different superscripts differ significantly.

^b^ Mean values in a row with different superscripts differ significantly.

* Significant at alpha = 0.05.

### 3.6 Correlation between DM, nutrient intakes, digestibility and weight gain

The results of correlation among DM, nutrient intake and digestibility, weight gain and SBW are presented in Table 8. The finding elucidated DM intake was positively and strongly correlated (*P* < 0.001) with OMI, CPI and SBW. DMI was also significantly correlated (*P* < 0.01) with CP digestibility. In addition, DM, OM and NDF digestibility and ADG were significantly correlated (*P* < 0.05) with dry matter intake for Gumuz lambs. The positive correlation between these factors indicates the enhanced fermentation and passage rate, which leads to improved intake because of the dietary treatments. Besides, the molasses offered to lambs might have contributed to the improved intake of DM and enhanced rumen fermentation and passage rate of supplement feed and basal diets. But NDF intake and ADF digestibility were no significantly correlated, but ADF intake was negatively correlated with DM intake. The CP intake was positively and significantly (*P* < 0.001) correlated with DM, OM, CP, NDF digestibility and SBW, which might be associated with the fact that increasing CP intake linearly increases CP digestibility, enhance microbial population and facilitates rumen fermentation. The CP digestibility was positively (*P* < 0.05) correlated with DM, OM and CP intake, and digestibility of DM, OM and ADFD (*P* < 0.05). In the current study, DM digestibility was strongly (*P* < 0.001) correlated with OM, CP digestibility. Similarly, the DM digestibility was significantly (*P* < 0.01) correlated with CP, NDF and ADF digestibility. The CP digestibility was significantly (*P* < 0.05) correlated with NDF, ADF digestibility and SBW. This indicates that as CP digestibility increases, the NDF digestibility and ADG also increase. This might be due to the effect of molasses, which cause improved fermentation in the rumen of animals by improving bacterial multiplication. The CP digestibility was highly significantly correlated (*P* < 0.001) with ADG and SBW, but negatively correlated with ADF digestibility. This might be because lignification of plant material hampers rumen fermentation of roughage feeds.

**TABLE 8:** Effect of replacement of NSC with different levels of CSH on correlation between dry matter and nutrients intake, digestibility and carcass characteristics of Gumuz yearling lambs fed MTNPH as a basal diet

	OMI	CPI	NDFI	ADFI	DMD	OMD	CPD	NDFD	ADFD	ADG	SBW
DMI	0.99***	0.77***	0.04^NS^	−0.15	0.43*	0.46*	0.57**	0.48*	0.26^NS^	0.42*	0.7***
OMI	1	0.77***	0.04^NS^	−0.15	0.43*	0.47*	0.57**	0.49*	0.25^NS^	0.42*	0.7***
CPI		1	−0.29	−0.44	0.76***	0.8***	0.8***	0.74***	−0.04	0.63**	0.76***
NDFI			1	0.42*	−0.29	−0.27	−0.51	−0.39	0.46*	−0.68	−0.26
ADFI				1	−0.52	−0.52	−0.45	−0.4	0.18^NS^	−0.44	−0.39
DMD					1	0.95***	0.73***	0.59**	0.02^NS^	0.51**	0.58**
OMD						1	0.76***	0.68**	0.04^NS^	0.56**	0.6**
CPD							1	0.67**	−0.32	0.72***	0.72***
NDFD								1	0.12^NS^	0.57***	0.46*
ADFD									1	−0.29	−0.16
ADG										1	0.14^NS^
SBW											1

Abbreviations: ADFD, acidic detergent fiber digestibility; ADFI, acidic detergent fiber intake; CPD, crude protein digestibility; CPI, crude protein intake; DMD, dry matter digestibility; DMI, dry matter intake; NDFD, neutral detergent fiber digestibility; NDFI, neutral detergent fiber intake; NS, non‐significant; OMD, organic matter digestibility; OMI, organic matter intake; SBW, slaughter body weight.

*** Significant correlation at alpha 0.001.

** Significant correlation at alpha 0.01.

* Significant correlation at alpha 0.05.

### 3.7 Partial budget analysis

The result of the partial budget analysis for the performance of Gumuz lamb on replacement of NSC with CSH fed on NPH basal diet is indicated in Table 9. The prices of feed ingredients used in this research were 0.5, 1.5, 5 and 10 ETB/kg for NPH, CSH, molasses and NSC, respectively. There was a substantial variation among treatments on a partial budget of replacement of NSC with CSH for Gumuz lambs. There was no loss of price per lamb in all treatments, and this might be due to the weight gain shown by experimental animals and better intake and good CP and energy content of experimental feeds during the trial period. Lambs on T5 had the highest net return compared with the other supplemented groups which fetch the net profit of 330.49 ETB/head; this might be due to the lower cost of CSH and better weight gain of lambs. The growth of net return and ΔNR in a high level of CSH substitution for NSC could be partly attributed due to the difference in selling price of lambs and mainly lower cost of CSH. The marginal rate of return (MRR) decreased as the level of CSH supplementation increased up to 313.79 g but increased at T5 (417.98 g) CSH. The increment in MRR in T5 might be due to the lower price of SH compared with the current high price of NSC. The current MRR result shows that each additional unit of 1 ETB per animal cost increment gain 0.5, 0.1 and 2 ETB profits for T2, T3 and T5 but lost −59.2 ETB in (T4), respectively. The higher negative value of MRR in T4 might be due to the addition of NSC in the treatment increased the total variable cost of the treatment and also lower selling price of lambs in T3 and T4.

**TABLE 9:** The partial budget analysis of experimental feeds

Variables	Treatments				
	T1	T2	T3	T4	T5
Purchasing price of sheep (ETB)	1552	1560	1470	1480	1520
Total NPH consumed (kg/animal)	320.4	317.9	317.7	309.7	301.9
Total CSH consumed (kg/animal)	0	50.4	100.8	151.2	201.6
Total molasses consumed (kg/animal)	6.53	5.71	4.9	4.07	3.66
Total NSC consumed (kg/animal)	127.8	95.85	63.9	31.95	0
Total cost of NPH (ETB/animal)	32.04	31.79	31.77	30.97	30.19
Total cost of CSH (ETB/animal)	0	10.08	20.16	30.24	40.32
Cost of molasses and medicine (ETB/lamb)	31	27.8	24.6	21.2	19
Total cost of NSC (ETB/animal)	255.6	191.7	127.8	63.9	0
Labor cost (ETB/animal)	360	360	360	360	360
Total variable cost (ETB/animal)	678.64	621.37	564.33	506.31	449.51
Selling price of sheep (ETB/animal)	2360	2283.3	2146.7	2196.7	2300
Total return	808	723.3	676.7	716.7	780
Net return	129.36	101.93	112.37	210.39	330.49
Change in NR	0	−27.43	−16.99	81.03	201.13
Change in TVC	0	−57.27	−114.31	−172.33	−229.13
MRR%	0	0.5	0.1	−59.2	2.0

Abbreviations: ETB, Ethiopian birr; MRR, marginal rate of return; TR, total return; ΔNR, change in net return; ΔTVC, change in total variable cost.

## 4 DISCUSSION

### 4.1 Chemical composition of the experimental feeds and refusals

The DM content of NPH offered in this study was almost comparable with values 91.3 and 91.6% reported by Gulilat et al. (2018) and Yigzaw et al. (2019), respectively. However, the current value is lower than the values 94.53 and 95.75% reported by Kibrom (2017) and Getent et al. (2019), respectively. Regarding findings in Ethiopia, the current CP value is higher than reported by Asmare et al. (2016) and Gelgelo et al. (2017), 3.5 and 3.73%, but lower than the value 9.81% reported by Gebregiorgis et al. (2017), respectively. The CP content of NPH used in the current study was below the minimum level (7%) required for maintenance in ruminant animals (Van Soest 1994). The differences in CP content among the various studies could be attributable to environmental factors in which the hay was grown, harvesting time and stage of growth at which the hay was harvested, drying process and grass–legume proportion of hay used for the experiment (McDonald et al., 2010; Wu, 2018).

The current NDF and ADF value of NPH was slightly comparable with the previous result of Yigzaw et al. (2019) and Gulilat et al. (2018), who reported 58.68 and 55.88% of NDF and 41.2 and 48.95% of ADF, respectively. However, these were lower than the values 71.23 and 83.85% of NDF and 46.43 and 48.98% of ADF contents reported by Asmare et al. (2016) and Kibrom (2017), respectively. In the feed refusals and offered composition, the NDF, ADF and ADL of refusal values were higher than the hay offered. This might be because experimental animals selected more edible portions of the basal diet and left the more fibrous parts (such as stems) of the grass which has higher fiber (NDF, ADF and ADL) fractions (Faji et al., 2019; Mekonen et al., 2015). This entails that when sheep are allotted in roughage diets, selection can be ensured through provision of adequate amount of feed for producers in tropical regions of the world.

The OM content of NSC used in this experiment is higher than the result 88.1 and 84.6% reported by Kebede (2014) and Galgileo et al. (2017), respectively. The current CP value of NSC (36.5%) was almost comparable with the earlier reports such as 36.2 and 34.65% by Ayele et al. (2017) and Yigzaw etal. (2019), respectively, in a different part of the country. However, the result is higher than that previously reported by Desta et al. (2017) and Gulilat et al. (2018) at 28.2; 33.4 and 30.57% of the CP content of NSC. But, lower than the 40% value reported by the Negussie et al. (2015) study conducted in the different part of the country. The difference in DM, OM and CP content of NSC observed by different investigators might be due to the variation of soil fertility crop grown, the amount of fertilizer applied, the stage of crop harvested and the method of oil extraction followed. McDonald et al. (2010) stated that the method of oil extraction causes variation in the nutrient content of products from oilseed cakes.

The OM contents of CSH used in the current experiment is lower than the value 90% reported by Tarekegn et al. (2018). The current CP value was higher than the value 16.69 and 17.15% reported by Abebe and Tamir (2016) and Gebrekidan et al. (2018), respectively. However, the current result is lower compared with the result of Agza et al. (2012), Etana et al.(2013) and Medikisa et al. (2016) who reported 24.42, 20.3 and 21.03%, respectively. Despite there exists variation in the composition of cowpea Sewinet variety (CSH) in different parts of Ethiopia, based on the classification of Lonsdale (1989), CSH used in the current experiment can be classified as a medium protein source feed for ruminant animals. In the present study, NDF content of CSH is lower than the value 57.77, 47.38% of NDF and almost similar value of ADF that 31.11 and 31.42% noted by Medikisa et al. (2016) and Tarekegn et al. (2019), respectively. This might be due to lower NDF content of CSH used in the current experiment showed that the hay is highly consumed and subsequent utilization by lambs (McDonald et al., 2010; Wu, 2018).

Generally, DM, OM and CP content of all experimental feed offered was greater than feed refusal while in T2, T3 and T4 the NDF, ADF and ADL percentage was the reverse. The reciprocal relationship between the protein and fiber content of plants was due to the maturity of the cell wall constituent along with lignin increase (McDonald et al., 2010). This might be due to the selective consumption behavior of the sheep. Refusals mainly constituted stem parts of the feed because the stem of the CSH must have been containing higher lignin content. The CP content of CSH offer was higher than refusal, but the NDF, ADF and ADL were contrary to CP content.

### 4.2 Dry matter and nutrient intakes

The basal diet DM intake of T5 was higher than the value (358 g/day) reported by Ayenew et al. (2019) in the Gumuz sheep breed supplemented with Camel's Foot Tree (*Piliostigma thonningii*) leaf meal and lower than the value (586.39 g/day) reported by Getent et al. (2019) for NPH supplemented with toasted soybean grain for the same breed. It has been learned that during the feeding experiment, the acceptance of CSH by experimental sheep was very high, which in turn elucidates the remarkable palatability of the hay. Moreover, the relatively low CP content of CSH compared with NSC that in turn forced lambs to consume more DM to meet their nutritional requirements. In line with this, McDonald et al. (2010) explained that in ruminant animal feeding, legume supplement creates a favorable rumen environment for microbial growth and increases the rate of feed digestion. Abebe and Tamir (2016) reported that higher improvement in total DM intake in Wollo lambs supplemented with pigeon pea; cowpea and lablab hay meals over with wheat bran and fed natural grass hay as a basal diet. The total DM intakes of the current result are higher than the value (569 g/day DM intake) reported by Ayenew et al. (2019) for Gumuz lambs supplemented sole Camel's Foot Tree (*Piliostigma thonningii*) leaf meal.

The OM, CP, NDF, ADF and ADL intakes are comparable with the range value (118.3–98.6 g/day) reported by Hailecherkos et al. (2020) for Washera sheep supplemented with different levels of Tree Lucerne (*Chamaecytisus palmensis*) fed Desho grass as a basal diet. Higher than the value 54.5, 54 and 84.8 g/day reported by Mekuriaw and Asmare (2018) and Ayenew et al. (2019); for Washera and Gumuz lambs sheep breed supplemented with graded levels of *Ficus thonningii* (Chibha) leaves, cowpea hay and Camel's Foot Tree (*Piliostigma thonningii*) leaf meal, respectively. The variation of CP intake in the current study from the previous findings might be due to the bulky nature of diets, sheep breeds, feeding method and environment in which experiments were conducted. The estimated ME intake of sole CSH supplemented sheep disagree with the previous finding of Gebrekidan et al. (2019). The ME requirement for a 20 kg lamb gaining 50–150 g/day is 3.7–6.4 MJ/day for diets with 65% of metabolizability (McDonald et al. 2010). According to ARC (1980), ME requirement for the maintenance and growth (50–200 g/day) gain for the same weight lamb is 4.5–7.9 MJ/day. Thus, based on these assumptions, the estimated ME of the treatment diets (5.7–6.68 MJ/day) in the current study was assured not only the maintenance but also the energy requirement as well as the growth of the experimental lambs was met. The increase in ME intake might be associated with the higher OM content in CSH. The current result was almost comparable with the finding of Hailecherkos et al. (2020), who reported a range of values from 7.3 to 5.2 MJ/day fed mixtures of Tree Lucerne (*Chamaecytisus palmensis*) dried leaves and concentrate mixture at different ratios for Washera lambs. However, lower than the result of Kibrom (2017) and Gebrekidan et al. (2019) the value from (6.51–11.82 MJ/day) fed different levels of concentrate mixture and (6.74–8.17 MJ/day) fed on wheat bran, CSH and its mixture for Begait sheep, respectively. The inconsistency of reports might be related to the climate variability, the type of basal diet and the difference in sheep breeds used for experiment.

### 4.3 Dry matter and nutrient digestibility

The DM and OM digestibility of the current study was lower than the value of Girmay and Amare (2017) who reported that 73.21 and 74.36% of digestibility for Abergelle goats supplemented with cowpea hay, respectively. But the current finding is greater than the value reported by 64% of DM and OM digestibility by Gelgelo et al. (2017) for black head Somali sheep supplemented with local brewery by‐product. This disparity might be due to the difference among sheep breed and the type of supplement feed used for respective experiments. The apparent digestibility of CP in this study is lower than the value 69 and 71.9% reported by Ayenew et al. (2019) and Getent et al. (2019) for Gumuz lambs supplemented with Camel's Foot Tree (*Piliostigma thonningii*) leaf meal and toasted soybean grain, respectively. The inconsistency of CP digestibility in different studies might be attributed to the feed preparation and processing methods and the difference of sheep breeds used in respective trials.

Higher apparent digestibility of ADF and NDF was observed in T5, associated with the lower fiber content of CSH, which was attained because of the optimum stage of maturity when forage was harvested. A study by Jung and Allen (1995) indicated that when the maturity of plants increases the digestibility decreases. The current value was lower than 69.69 and 62.71% of NDF and ADF value reported by Tarekegn et al. (2019) for Abergelle goat supplemented with cowpea hay. The inconsistency of findings might be related to the nature of feeds and processing methods, feeding practices and species and breeds of animals used in different experiments. The higher DM, OM, NDF and ADF digestibility observed for CSH supplemented groups in the current study might be due to high feed intake and legume forages are rich in nitrogen that facilitates fermentation of roughage feeds in ruminants. The study by Getent et al. (2019) indicated that DM intake was positively correlated with OM, CP and ADF intake and digestibility. McDonald et al. (2010) indicated that improvement in DM and OM digestibility associated with legume forage leaf meal is due to better nitrogen access in the rumen, thereby improving the rate of fermentation of ingested feed.

### 4.4 Body weight change, daily gain and FCE

The current finding in ADG is higher than the value 20.33 g reported by Abebe and Tamir (2016) for Wollo sheep supplemented with a mixture of 260 g cowpea hay with 200 g wheat bran and 32.2 g by Tiruneh et al. (2019) for local sheep supplemented with 588 g pigeon pea‐dried leaves The average value of the current finding is lower than the value 64.44 g by Yigzaw et al. (2019) for Farta sheep supplemented 400 g lentil hull and 50 g NSC. The difference observed among various findings for this parameter might be due to the type of sheep breeds, supplement used and the difference in soil type, harvesting time of basal diet used for experimental animals. Replacement of NSC up to 50% with CSH showed to reduce ADG and FCE, but at 75 and 100% replacements appeared to be a better option for inclusion in the growth of sheep. This indicates that higher level of CSH supplementation with concentrate mixture has flourished a better impact on lambs’ overall performance.

The FCE observed in the present study is higher than the previous report 0.031 FCE reported by Abebe and Tamir (2016) for Wollo sheep supplemented with a mixture of SH with wheat bran and the value 0.053 by Gebrekidan et al. (2019) for Begait sheep supplemented with cowpea hay. It was lower than the value 0.08 reported by Getent et al. (2019) for Gumuz lambs supplemented with toasted soya bean grain. This variation may be due to the difference in type of supplement feed used for the experiment, DM, CP and ADF intake and digestibility of lambs, the type of birth and birth weight of lambs. The performances of Gumuz sheep for FBW, ADG and FCE in sole NSC and at an increasing level of CSH supplementation were higher compared with other treatments. This might be due to the higher CP content of NSC and the high nutrient digestibility of the sole SH diet due to legume forages improve rumen micro‐organism.

### 4.5 Slaughter and EBW, HCW and DP

The current finding agrees with the value 17.3 kg EBW and 9.25 kg HCW reported by Gelgelo et al. (2017) for black head Somali sheep supplemented with local brewery by‐product and concentrate mixture. However, these values were lower than those reported by Getent et al. (2019) at 24.17 kg and 9.72 kg EBW and HCW for Gumuz lambs supplemented with toasted soybean grain, respectively. The disparity of current results from previous findings might be associated with the variation on CP digestibility, FCE of lambs and also the breed of sheep used for the experiment. For parameters of DP on EBW base is higher than the values (33%) reported by Lamaro et al. (2016) for Hararghe highland sheep supplemented maize stover with concentrate mix. On the other hand, these values were lower DP than the value (48%) reported by Tesfay et al. (2017) for Tigray sheep consumed mulberry leaf meal with a mixture of concentrate mixture, (45.22%) by Kibrom (2017) for Begait sheep supplemented with different levels of concentrate mixture, (40.69%) reported by Getent et al. (2019) supplemented with toasted soybean grain and (46.9%) supplemented with treated local bamboo leaf hay for Gumuz lambs. The inconsistency of results might be due to the nutrient content of experimental diet, DM, CP intake and digestibility, the weight gain of lambs, the breed difference used for the trials. The DP on an EBW basis of the current result was nearly comparable with the value (50.17%) reported by Tsega et al. (2019) for Menz lambs supplemented with wheat bran and higher than (44.2%) reported by Lamaro et al. (2016) for Hararghe highland sheep but lower than the value (51.18%) reported by Getent et al. (2019) for Gumuz lambs. This study achieved higher DP on SBW and EBW base at a higher level of NSC and CSH supplementation. This improvement might be due to the higher CP content of NSC and higher DM digestibility of sole Sewinet hay–supplemented group that enhanced muscle development of lambs.

### 4.6 Rib‐eye muscle area

The current study concurs well with the value (7.33 cm^2^) reported by Getent et al. (2019) for Gumuz lambs supplemented with toasted soybean grain for Gumuz lambs. Higher than the value (4.99 cm^2^) reported by Ayenew et al. (2019) for Gumuz lambs supplemented with Camel's Foot Tree (*Piliostigma thonningii)* leaf meal. But lower than the value (9.9 and 8.93 cm^2^) reported by Tesfay et al. (2017) for Tigray highland lambs supplemented with a mixture of 150 g concentrate mix with 173.1 g mulberry leaf meal groups and Desta et al. (2017) for Abergelle lambs on replacement of dried mulberry leaf meal for concentrate mix. The inconsistency of different reports might be due to variations in the type of basal diet, the total DM intake, the weight gain, FCE breeds used for those experiments.

### 4.7 Carcass offal

The amount of carcass offal obtained in the current result is higher than value 64.2 and 90 g reported by Adugna et al. (2020) and Hailecherkos et al. (2020) for Gumuz and Washera lambs supplemented with treated local bamboo leaf hay and different levels of Tree Lucerne (*Chamaecytisus palmensis*) fed Desho grass as a basal diet, respectively. This variation might be due to the difference of NDF and ADF content, DM digestibility and the weight gain of lambs. The value of the current finding is in contradiction with the value reported by Gelgelo et al. (2017) for Somali sheep supplemented with local brewery by‐product (*Tata*), and concentrate mix significantly improved the total weight of edible offal such as blood, heart, liver, tongue, reticulo‐rumen and tail. The result was higher than the value (3.31 kg) described by Gulilat *et al*. (2018) for Farta sheep supplemented with concentrate but lower than the value 5.3 and 4.3 kg reported by Tesfay et al.(2017) for Tigray highland lambs supplemented with mulberry leaf meal and Getent et al. (2019) for Gumuz lambs supplemented with toasted soybean grain. This variation might be due to the difference in type of sheep breeds, total dry matter intake, CP content of the basal diet, and the DM digestibility and daily gain of lambs used for the experiment.

Unlike the current result, studies conducted by Gemechu and Mekash (2015) for Arsi‐bale sheep, Tesfay et al. (2017) for Tigray highland lambs on a different level of mulberry (*Morus alba*) leaf meal reported a significant difference in TNEO weight and Negewo et al. (2019) for local sheep supplemented on graded levels of concentrate mix. The values reported were same to the previous finding of Yirdawet al. (2017) for Bageit sheep supplemented with Cottonseed cake, dried *Acacia saligna*, *Sesbania sesban* and *Vigna unguiculata* supplements, and Getent et al. (2019) for Gumuz lambs supplemented with toasted soybean grain was reported non‐significant difference on TNEO. Variations observed might be related to breeds of lambs used, feed types and management of animals during feeding in respective experiments.

### 4.8 Correlation between DM, nutrient intakes, digestibility and weight gain

In the current study, as CP intake increased, DM intakeand digestibility also increased, which in turn enhanced ADG and SBW. This is similar to the result of Mekonnen et al. (2019) who indicated that the increased in DM intake was increased DM digestibility of the ration and improved body weight changes of animals. The current result was similar to the report of Adugna et al. (2020) and Hailecherkos et al. (2020), who indicated that CP intake was positively correlated with intake of DM, OM and CP and OM and CP digestibility and ADG of CP, but not correlated with NDF and ADF digestibility. The positive association of CP digestibility with NDF, ADF digestibility and SBW might be due to the effect of molasses, which cause improved fermentation in the rumen of animals by improving bacterial multiplication. White et al. (1973) reported that the addition of molasses (0–20%) to a straw‐based diet significantly increases both DM and crude fiber digestibility. The CP digestibility was highly significantly correlated (*P* < 0.001) with ADG and SBW, but negatively correlated with ADF digestibility. This might be due to lignification of plant material that hampers rumen fermentation of roughage feeds.

### 4.9 Partial budget analysis

The growth of net return and ΔNR in a high level of CSH substitution for NSC could be partly attributed due to the difference in the selling price of lambs and mainly lower cost of Sewinet hay. The current result was comparable with the value 338.49 ETB reported by Desta et al. (2017) for Abergelle sheep on 75% of substitution of mulberry leaf meal for concentrate mix. Although it is higher net return in sole Sewinet hay than 185.63 ETB reported by Kibrom (2017) for Begait sheep at a different level of supplementation, 230.7ETB by Yigzaw et al. (2019) for Farta sheep on 400 g of Lentil Hull with 50 g NSC that indicated higher net return and 240.12 ETB by Mekonnen et al. (2019) for Washera sheep supplemented with 50% CM and 50% hydroponically grown barley and oats forage. This was lower than the value 740 ETB reported by Hailcherkos et al. (2020) for Washera sheep supplemented with Tree Lucerne dried leaf.

Regarding MRR, an increment was observed in T5 which might be due to the lower price of SH compared with the current high price of NSC. The current MRR result shows that each additional unit of 1 ETB (0.025 USD) per animal cost increment gain 0.5, 0.1 and 2 ETB profits for T2, T3 and T5 but lost −59.2 ETB in (T4), respectively. The higher negative value of MRR in T4 might be due to the addition of NSC in the treatment increased the total variable cost of the treatment and also lower selling price of lambs in T3 and T4. The current result was similar to Meaza (2012) for Tigray highland rams on supplemented with graded levels of mixtures of a sesame seed (*Sesame indicum)* cake and wheat bran marginal rate of return was decreased but the net return was inversed abovementioned parameter. As a result, based on the outcome of the current study, it is not necessary to spend additional expenses for high‐price concentrate feeds T5 (417.98 g), CSH could be considered as an economically feasible supplement for growing lambs compared with T1, T2, T3 and T4.

## 5 CONCLUSION AND RECOMMENDATION

The current study revealed that inclusion of CSH in a native hay‐based diet helped to meet the CP maintenance requirement and for muscle development of lambs, indicating the need for supplementation of protein source feed for body weight gain. Therefore, based on the present result, supplementation of 417.98 g of CSH (T5) had increased the total DM and nutrient intake and carcass yield of lambs superior to sole NSC supplementation. In terms of economic returns and the biological performance of lambs, the dietary CSH supplementation in the treatment group (T5) was highly recommended. To confirm feasibility of the finding, it is vital to conduct an on‐farm experiment under farmer's management for effective utilization of the results.

## CONFLICT OF INTEREST

The authors declare that they have no conflict of interest.

## AUTHOR CONTRIBUTION

All the authors contributed in research conceptualization, experimental design, data collection and data curation, analysis and final draft manuscript writing.

## Consent for publication

All authors agreed on the publication of this paper and assigned corresponding author responsible in charge for correspondence during manuscript publishing.

### PEER REVIEW

The peer review history for this article is available at https://publons.com/publon/10.1002/vms3.617

## Data Availability

Data is available with the corresponding author upon request
